# Rethinking Potassium Management in Chronic Kidney Disease—A Modern Approach

**DOI:** 10.3390/jcm14248917

**Published:** 2025-12-17

**Authors:** Zheng Xi Kog, Ivan Wei Zhen Lee, Swee Ping Teh

**Affiliations:** 1 Department of Renal Medicine, Singapore General Hospital, Singapore 169608, Singapore; kog.zheng.xi@singhealth.com.sg; 2 Department of Renal Medicine, Sengkang General Hospital, Singapore 544886, Singapore; ivan.lee.w.z@singhealth.com.sg

**Keywords:** hyperkalemia, chronic kidney disease (CKD), dietary restriction, renin-angiotensin aldosterone system (RAAS) inhibitors

## Abstract

Potassium homeostasis is impaired in patients with chronic kidney disease (CKD) due to alterations in physiological mechanisms and use of agents that modulate the renin angiotensin aldosterone system (RAAS) to slow CKD progression and reduce cardiovascular risk. In recent years, a new paradox has emerged: while dietary potassium restriction has been dogmatically recommended to prevent hyperkalemia, emerging evidence suggests that a more liberated potassium intake may offer potential benefits, particularly in patients with early CKD. This has prompted a paradigm shift towards a more individualized approach to the management of hyperkalemia in CKD. This review aims to provide an overview of the latest management strategies for hyperkalemia in CKD and to summarize the current literature including publications and guidelines recommendations with respect to dietary potassium intake and use of potassium salt substitutes.

## 1. Potassium Homeostasis in Patients with Chronic Kidney Disease

### 1.1. Normal Potassium Homeostasis

Potassium is a predominantly intracellular cation, with approximately 2% (~60–80 mmol) of total body potassium (~3000–4000 mmol/L) found in extracellular fluid and the remaining 98% located intracellularly. Plasma potassium concentrations are maintained within a narrow range from 3.5–5.0 mmol/L by (i) matching potassium intake with excretion and (ii) ensuring proper distribution between extracellular and intracellular compartments. Potassium is primarily excreted by the kidneys, while several mechanisms govern its transmembrane distribution [1].

Insulin, whether secreted in response to a glucose load or administered exogenously, promotes an intracellular shift of potassium by binding to specific cell-surface receptors. This leads to the insertion of Glucose Transporter type 4 (GLUT4) and facilitates glucose uptake in insulin responsive tissues. Insulin also stimulates potassium uptake by increasing the activity of the adenosine triphosphatase sodium/potassium pump (Na^+^/K^+^-ATPase). In patients with CKD, insulin-mediated glucose uptake is impaired, but potassium shifts remain largely intact, suggesting subtle differences in the regulation of insulin-mediated glucose and potassium uptake.

Catecholamines bind to β_2_ receptors, enhancing Na^+^/K^+^-ATPase activity and thereby limiting increases in extracellular potassium. Elevated interstitial potassium concentrations induce vasodilation, increasing skeletal muscle blood flow. In contrast, total-body potassium depletion or hypokalemia reduces skeletal muscle perfusion, contributing to the association between hypokalemia and rhabdomyolysis.

Changes in plasma tonicity and acid-base disorders also influence transcellular potassium distribution. An increase in extracellular osmolality creates an osmotic gradient that drives water out of cells, raising intracellular potassium concentration of potassium and promoting potassium efflux through K^+^ permeable channels. Acidemia, particularly hyperchloremic normal anion gap metabolic acidosis, reduces the transmembrane rate of Na^+^/H^+^ exchange via the Na^+^/H^+^ exchanger (NHE1) and intracellular shift of HCO_3_^−^ via Na^+^/HCO_3_^−^ cotransporter 1 and 2 (NBCe 1 and 2). This leads to a reduction in intracellular sodium, decreased Na^+^/K^+^-ATPase activity, and diminished potassium influx. Concurrently, lower extracellular HCO_3_^−^ increases Cl^−^ influx by Cl^−^/HCO_3_^−^ exchange, while reduced intracellular HCO_3_^−^ promotes Cl- efflux, both contributing to potassium efflux via K^+^/Cl^−^ cotransport.

In the kidney, potassium is freely filtered by the glomerulus and ~90% reabsorbed in the proximal tubule (paracellularly) and the ascending loop of Henle via the apical membrane Na^+^/K^+^/2Cl^−^ cotransporter (NKCC). This resorptive component of kidney potassium handling is largely independent of dietary potassium intake. Potassium secretion primarily occurs at the Aldosterone-Sensitive Distal Nephron (ASDN), which includes the Distal Convoluted Tubule (DCT), collecting ducts. This is mediated by two types of apical K^+^ channels and a negative polarized luminal fluid maintained through Na^+^ reabsorption via apically-located epithelial Na^+^ channels (ENaC) [2]. Aldosterone stimulates ENaC activity through mineralocorticoid receptors, increasing the number of ENaC and the proportion of time the channel remains open [3]. Potassium is then secreted into the lumen through the renal outer medullary potassium channels (ROMK; also referred to as Kir 1.1) in principal cells, and BK channels in principal and intercalated cells of the collecting duct. The latter is upregulated by increases in fluid flow rate, accounting for the flow dependent component of distal potassium secretion. This secretory component of kidney potassium handling is governed by dietary intake, and is influenced by various factors including luminal Na^+^ delivery and flow rate, plasma potassium concentration, circulating aldosterone and arginine vasopressin levels and acid-base status [4].

With high dietary potassium intake, increased distal Na^+^ delivery and tubular flow to the ASDN enhance potassium excretion. Elevated plasma potassium concentration is sensed by basolateral Kir4.1/5.1 channels at the initial portion of the DCT (DCT1), modulating the activity of WNK family and their downstream effectors of SPAK and OxSR1. The reduces Na^+^/Cl^−^ cotransporter (NCC) activity, increasing distal sodium delivery and flow to the later DCT (DCT2) and the collecting ducts, thereby promoting potassium secretion. Chronic high potassium intake further amplifies this effect via reduced Na^+^ reabsorption in the thick ascending limb and proximal tubules, due to medullary recycling and interstitial potassium accumulation. This natriuretic effect of a high dietary potassium effect is postulated by Palmer et al. have proposed that this natriuretic effect may underlie the blood pressure lowering benefits of salt substitutes, especially in individuals with high dietary Na^+^ intake [1].

Conversely, low dietary potassium intake reduces plasma potassium levels, increases NCC activity in DCT1, and limit potassium secretion by decreasing Na^+^ delivery and flow to the ASDN. This process has been implicated in the pathogenesis of salt-sensitive hypertension.

Additional regulatory mechanism includes (i) an enteric potassium-sensing pathway and (ii) circadian variation in potassium transporters expression. The splanchnic sensing mechanism detects potassium entry into the gastrointestinal tract and suppresses NCC activity via dephosphorylation independent of plasma potassium or mineralocorticoid levels. Potassium excretion follows a circadian rhythm- lower during the night and early morning, and higher during the day, aligning with dietary potassium intake. This pattern is attributed to diurnal variations in ROMK gene expression, which peaks during daylight and activity periods, while H^+^/K^+^ ATPase expression follows an inverse pattern [1].

### 1.2. Impaired Renal Excretion

Insult to the ASDN from various forms of kidney injury such as acute tubular necrosis in acute kidney injury (AKI), tubulointerstitial disease or obstructive uropathy, can impair potassium excretion. For example, in patients with obstructive uropathy, renal injury begins as a result of elevated intratubular pressure, leading to tubulointerstitial inflammation and finally structural changes (fibrosis and tubular cell apoptosis) with impairment of tubular function. This includes (i) impaired sodium excretion due to downregulation of sodium channels in the ASDN including Na^+^/K^+^ ATPase, NHE1 and sodium-hydrogen exchange 3 (NHE3) and (ii) impaired urinary acidification contributing to the development of metabolic acidosis [5,6,7]. Additionally, acute reductions of glomerular filtration rate (GFR) lead to decreased distal sodium delivery and tubular fluid flow, further reducing potassium secretion. Other contributing mechanisms include metabolic acidosis, increased catabolism, and tissue breakdown, which promote transcellular shift or release of intracellular potassium into the extracellular compartment.

In patients with CKD, hyperkalemia is uncommon when GFR > 60 mL/1.73 m^2^ and increases but increasingly prevalent as GFR declines [8]. In early CKD, potassium homeostasis is preserved through adaptive increase in potassium secretion in the ASDN, akin to the physiological response seen in healthy individuals on a high potassium diet [1]. This functional adaptation is accompanied by structural changes, including cellular hypertrophy, increased mitochondrial density and proliferation of the basolateral membrane in cells in the distal nephron and principal cells of the collecting duct [1]. Elevated serum potassium and mineralocorticoid activity independently initiate this process, which is accompanied by an increase in Na^+^/K^+^ ATPase activity. Enhanced distal sodium delivery and tubular fluid flow to the ASDN in remaining functional nephrons also help to maintain the net potassium secretion.

As renal function declines, gastrointestinal potassium secretion, particularly in the colon, becomes increasingly important in maintaining total body potassium [1]. This process involves increased potassium uptake across the basolateral membrane and secretion into the intestinal lumen by the large-conductance Ca^2+^-activated (K_Ca_1.1) BK channel, regulated by aldosterone and other factors that elevate cyclic adenosine monophosphate in the enterocyte.

When GFR falls below 30 mL/1.73 m^2^, hyperkalemia gradually develops through several mechanisms

(i) Decreased distal Na^+^ delivery, as seen in decompensated heart failure or acute glomerulonephritis,
(ii) Reduced mineralocorticoid activity, common in diabetes with hyporeninemic hypoaldosteronism or due to RAASi therapy,
(iii) Abnormal collecting duct function, often associated with tubulointerstitial kidney disease) [1].

## 2. Management of Hyperkalemia in Chronic Kidney Disease

Hyperkalemia is a common electrolyte disorder in patients with impaired renal function, occurring in the context of AKI or advanced CKD, particularly in patients with GFR < 30 mL/min/1.73 m^2^ [9,10,11]. This is in partly due to the mechanisms previously described and is further exacerbated by pharmacological interventions aimed at slowing CKD progression, specifically, renin-angiotensin aldosterone system inhibitors (RAASi) including angiotensin-converting enzyme inhibitors (ACEi), angiotensin-receptor blockers (ARBs) and non-steroidal/steroidal mineralocorticoid receptor antagonists (ns-MRAs/MRAs) [12]. Other risk factors for hyperkalemia include elevated urine albumin-to-creatinine ratios, diabetes mellitus, heart failure, constipation and medication use (i.e., NSAIDs, COX2-inhibitors, sulfamethoxazole-trimethoprim, beta-blockers, ciclosporin and tacrolimus) [13,14].

### 2.1. Potential Implications of Hyperkalemia on Patients with Chronic Kidney Disease

#### 2.1.1. Hyperkalemia and Its Significance in Patients with CKD

There is no clear consensus for the definition of hyperkalemia in current guidelines and literature, it is generally defined as a serum potassium > 5.0–5.5 mmol/L. It can be further classified based on the degree of elevation from mild (serum potassium 5.1–5.4 mmol/L), moderate (serum potassium 5.5–5.9 mmol/L) to severe (serum potassium ≥ 6.0 mmol/L) [14,15].

Acute hyperkalemia can potentially result in life-threatening cardiac dysrhythmia and cardiac arrest [16]. Chronic dyskalemias, on the other hand, have been associated with adverse outcomes in patients with CKD such as a “U-shaped” pattern with mortality and increased risk of CKD progression [9,17,18,19]. A meta-analysis by Kovesdy et al. involving 27 international cohorts (10 chronic kidney disease included) and 1,217,986 participants, found that hyperkalemia was more prevalent among individuals with lower eGFR and higher albuminuria. Both hypo- and hyperkalemia were associated with increased risk of all-cause mortality [20].

Beyond its direct clinical consequences, hyperkalemia poses a significant barrier to the optimization of guideline-directed medical therapy. It often necessitates dose reduction or cessation of RAASi, thereby increasing the risk of adverse cardiovascular and renal outcomes, as well as healthcare utilization and costs [11,14].

Therefore, the management of hyperkalemia is of great importance, particularly in patients with high risk of CKD progression and attendant risks of cardiovascular morbidity and mortality. The 2024 Kidney Disease: Improving Global Outcomes (KDIGO) Guidelines for Evaluation and Management of CKD recommend a multipronged approach. This includes reviewing concurrent medications, moderation of dietary potassium intake, and considering adjunctive therapies such as sodium bicarbonate supplementation (in patients with metabolic acidosis), diuretics (in those with fluid overload) and potassium binders to enable continued RAASi therapy [13].

#### 2.1.2. Discontinuation/Dose Reduction of Renin-Angiotensin-Aldosterone System Inhibitors

A systematic review by Floege et al. identified numerous studies involving CKD patients with who required dose reduction or cessation of RAASi due to hyperkalemia (16 studies on CKD patients with sub-optimal RAASi dosing due to hyperkalemia and 44 studies on CKD patients with RAASi cessation due to hyperkalemia) [11]. These findings reflect common clinical practice, where prescription or optimization of RAASi dose is often limited by hyperkalemia, particularly in refractory hyperkalemia.

Numerous studies have reported a myriad of adverse cardiorenal outcomes associated with dose reduction or cessation of RAASi in patients with CKD (Table 1). These include major adverse cardiovascular events (MACE), cardiovascular mortality, all-cause mortality, CKD progression, increased risks of hospitalization and prolonged hospital stays, particularly among individuals with advanced CKD [19,21,22,23,24,25,26].

#### 2.1.3. Hyperkalemia-Related Healthcare Utilization, Costs

Given the clinical implications of hyperkalemia with CKD, particularly in patients who require dose reduction or cessation of RAASi prescription, it is unsurprising that affected individual patients with hyperkalemia tend to have higher healthcare utilization and incur greater costs, compared to patients with normokalaemia. A systematic review by Floege et al. identified numerous studies reporting that CKD patients with hyperkalemia were associated with higher healthcare utilization (in terms of greater average hospitalizations and longer hospital length of stay) and costs, regardless of CKD stage, compared to patients with normokalemia [11].

### 2.2. Traditional Strategies for Management of Hyperkalemia

#### 2.2.1. Correction of Metabolic Acidosis

A reduction in nephron mass in CKD leads to impaired net acid excretion (contributed by hyperkalemia), ultimately contributing to the development of chronic metabolic acidosis. In turn, metabolic acidosis promotes hyperkalemia by facilitating the transcellular shift of potassium from the intracellular to extracellular compartment in exchange of excess hydrogen ions [27]. Previous literature and clinical guidelines recommend routine sodium bicarbonate supplementation to maintain serum bicarbonate concentration above 22 mmol/L, aiming to prevent complications of chronic metabolic acidosis such as protein-energy wasting, insulin resistance, bone demineralization, and CKD progression [28,29,30]. The latest 2024 KDIGO guidelines have since revised this recommendation and instead suggest prescription of sodium bicarbonate only to avoid severe metabolic acidosis (serum HCO_3_^−^ < 18.0 mmol/L) in adults with CKD [13,14]. However, the guidelines were vague with respect to their stepwise approach to managing hyperkalemia (serum potassium > 5.5 mmol/L) and did not specify the means or a treatment target when suggesting optimization of serum bicarbonate levels [13]. Given the costs of novel potassium binding resins, we believe that sodium bicarbonate supplementation might still offer a cheaper alternative to managing milder degrees of hyperkalemia by reducing acidemia and increasing sodium delivery to the ASDN. An alternative strategy in the management of metabolic acidosis involves increasing the consumption of fruits and vegetables [13]. This dietary approach is supported by several small randomized controlled trial, demonstrating comparable efficacy and safety to oral sodium bicarbonate in patients with CKD [31,32,33,34].

#### 2.2.2. Prescription of Diuretics

Loop or thiazide diuretics are commonly prescribed for the management of fluid overload or hypertension in patients with CKD. Loop diuretics are typically the preferred agents in advanced CKD, largely due to traditional belief and older guideline recommendations, based on weak evidence that thiazide diuretics are less effective when the GFR falls below30 mL/min/1.73 m^2^ [35,36]. However, a growing body of evidence, including a recent randomized, placebo-controlled trial involving 160 patients with stage 4 CKD and poor controlled hypertension, has demonstrated that thiazide-like diuretics, particularly chlorthalidone, can significantly reduce blood pressure in this population [37,38,39]. Both loop and thiazide diuretics require higher doses in advanced CKD to achieve a therapeutic effect and exhibit additive effect when used in combination, especially in the management of severe or refractory volume overload [37]. A common adverse drug reaction for these drugs includes hypokalemia, which may paradoxically be beneficial in managing concomitant hyperkalemia [38,39].

#### 2.2.3. Dietary Potassium Restriction

Historically, medical practitioners have recommended dietary potassium restriction of 2–3 g/day, despite a lack of strong evidence supporting this practice [40,41]. With increasing use of emerging medical therapies such as non-steroidal mineralocorticoid receptor antagonists to delay CKD progression [42,43], it remains common practice to advise patients to restrict dietary potassium, either with or without the use of novel potassium binding resins, in order to optimize the prescription of RAAS inhibitors [44,45,46]. However, a growing body of evidence highlights potential risks of this traditional approach with respect to CKD, adverse cardiovascular outcomes and all-cause mortality (Table 2) [47,48,49,50].

In animal models involving male rats with CKD (compared to controls with normal renal function), low potassium intake led to inflammation, nephromegaly and kidney function decline while the high potassium intake reduces arterial pressure, hyperaldosteronism and kidney fibrosis [51]. Additionally, potassium citrate attenuated the hypertensive effects of a high-potassium diet but did not mitigate its profibrotic impact. These findings suggest that in patients with CKD, both low and high extremes of dietary potassium intake may be harmful, underscoring the need for further research to determine the optimal intake range [51].

There is growing recognition within the nephrology community of the potential risks associated with routine dietary potassium restriction with emerging evidence supporting the cardiovascular benefits of higher dietary potassium intake suggesting that it is time to rethink our approach to potassium management in patients with CKD [52,53]. This shift is reflected in the 2024 update of the Kidney Disease Improving Global Outcomes CKD Guidelines, which advocate for a personalized approach that considers the patient’s CKD stage, comorbidities, quality of life, history of hyperkalemia, and sources of dietary potassium, with an emphasis on minimizing intake of avoiding highly processed foods [13].

### 2.3. Modern Strategies for Management of Hyperkalemia

#### 2.3.1. Role of Sodium Glucose Transporter 2 Inhibitors (SGLT2i)

SGLT2i induces both osmotic diuresis and natriuresis by inhibiting glucose and sodium reabsorption at the proximal tubule. This increases sodium delivery to the distal convoluted tubule and collecting duct which, thereby promoting potassium excretion. SGLT2i are a cornerstone of optimal medical therapy for patients with diabetes mellitus CKD and heart failure [54,55]. Notably, beyond their established benefits in reducing cardiovascular and renal adverse outcomes, emerging evidence also suggests that SGLT2i may lower the risk of hyperkalemia in at-risk populations.

A meta-analysis by Neuen et al., which included six studies involving 49,875 participants evaluated the use of SGLT2i in diabetic patients with high cardiovascular risk or with CKD and a showed 16% relative risk reduction of serious hyperkaliemia (serum potassium ≥ 6.0 mmol/L), even in the setting of concomitant RAASi prescriptioin [56].

A post-hoc analysis of the FIDELIO-DKD study by Agarwal et al. noted that combination therapy with SGLT2i and non-steroidal mineralocorticoid antagonists (nsMRA) finerenone was associated with a lower risk of hyperkalemia [43]. This finding was corroborated by the recent CONFIDENCE randomized, placebo-controlled trial by Agarwal et al. involving 784 diabetic patients with proteinuria, CKD on pre-existing RAASi comparing the use of SGLT2i or nsMRA monotherapy vs. combination therapy with SGLT2i and nsMRA [57]. Combination therapy with SGLT2i and nsMRA not only achieved greater reduction in urine albumin creatinine ratio (uACR) compared to either monotherapy but also demonstrated a lower risk of hyperkalemia than monotherapy with nsMRA [57].

These findings underscore the potential role of SGLT2i as a valuable adjunct in managing mild hyperkalemia, thereby facilitating the continued use or up-titration of RAASi.

#### 2.3.2. Prescription of K-Binding Resins

Historically, sodium polystyrene sulfonate (SPS) has been used to manage hyperkalemia. However, recent studies have raised concerns about its efficacy in addition to other reports of severe gastrointestinal adverse effects, most notably intestinal necrosis [58,59]. Safer and more effective therapeutic options like sodium zirconium cyclosilicate (SZC) and patiromer have emerged in recent years for treatment of acute and chronic hyperkalemia in various at-risk populations (Table S1).

Sodium zirconium cyclosilicate (SZC) is a highly selective cation exchanger that entraps potassium in the intestinal tract in exchange for sodium and hydrogen. It is administered as a tasteless, odorless powder mixed with water form an oral suspension, offering a more palatable alternative to the notoriously gritty and unpleasant-tasting SPS. In the treatment of acute hyperkalemia, 10 g of SZC three times a day lowers serum potassium by 0.4 mmol/L (95% CI −0.5 to −0.4) within 2 h, with a mean reduction of serum potassium by 0.7 mmol/L (95% CI −0.7 to −0.6; −12%) at 24 h. The potassium-lowering effect is dose-dependent, and patients with higher baseline potassium levels tend to experience greater reductions [60,61,62]. For chronic hyperkalemia, long-term use of SZC has been shown to be effective in maintaining normokalaemia in both CKD patients on RAASi without dietary restriction and ESKD HD population [63,64,65,66]. SZC is also effective in patients with heart failure to allow for maintenance or optimization of MRA dose [67]. Additionally, SZC has been associated with improvement in serum bicarbonate levels, with mean percentage increase of 4–6%, which may confer protective effect among individuals with CKD [63]. Furthermore, SZC has been shown to be a cost-effective treatment for hyperkalemia [68]. Common adverse events of SZC include hypokalemia, particularly during the initial correction phase, where it has been linked to QT interval prolongation, dose-dependent oedema, and gastrointestinal symptoms [61,63].

Patiromer is a non-absorbed polymer that binds potassium in exchange with calcium throughout the gastrointestinal tract, thereby enhancing fecal excretion and lowering serum potassium levels. It is effective in treating acute hyperkalemia in high-risk populations, including patients with CKD, heart failure, and diabetes mellitus, and was the first cation-exchanger after SPS to be approved by the Food Drug Administration (FDA). In the OPAL-HK trial, patiromer use while maintaining RAASi resulted in a mean reduction of serum potassium by 1.01 mmol/L at week four, with greater reduction observed in patients with moderate-to-severe hyperkaliemia. Notably, 76% of patients achieved normokalaemia by week 4 without dietary restriction with patiromer [69]. Long-term use over 52 weeks also demonstrated sustained reduction in serum potassium [70]. Additionally, short-term use over 4 weeks in a small cohort of hemodialysis patients demonstrated its efficacy in reducing the episodes of hyperkalemia [71]. Patiromer was generally well tolerated, with common adverse effects like gastrointestinal intolerance and hypomagnesaemia.

Given the efficacy and safety of novel potassium binding resins like SZC and Patiromer, recent guidelines have increasingly supported their use in patients with CKD and hyperkalemia to facilitate the optimization of RAAS inhibitor therapy and delay CKD progression have encouraged the increased use of these agents in patients with CKD and hyperkalemia to enable optimization of medical therapy such as RAASi to delay CKD progression [13,14].

## 3. Liberation of Potassium Restriction and Consideration of Potassium Supplementation/Salt-Substitutes

### 3.1. Evidence Demonstrating Potential Benefits of High Dietary Potassium Intake

A narrative review by Palmer et al. published in 2016 outlined several potential health benefits associated with high dietary potassium intake. These included reduction in blood pressure and stroke risk, decreased incidence of nephrolithiasis, favorable effects on bone health for the general population and potential improvement in metabolic acidosis and dietary phosphate absorption in patients with CKD [72]. Numerous studies have further strengthened the evidence base supporting the individual and public health benefits of a higher dietary potassium intake, particularly when combined with reduced dietary sodium intake, in improving blood pressure control and preventing the development cardiovascular disease and CKD (Table 3) [73,74,75,76,77].

In terms of cardiovascular outcomes, a notable study to highlight is an open-label, cluster-randomized trial across 600 villages in rural China, involving 20,995 individuals either aged over 60 years or with a history of stroke and poorly controlled blood pressure. The study demonstrated that using a potassium-based salt substitute, compared with regular salt, significantly reduced the risk of stroke, major adverse cardiovascular events and all-cause mortality, without an increase in serious adverse events attributed to hyperkalemia. However, it is important to note that the study excluded patients who self-reported any history of serious kidney disease and no baseline renal function assessments were performed [78]. These findings were corroborated by a systematic review and meta-analysis encompassing 21 studies and 31,949 participants, which reaffirmed the benefits of salt substitution in lowering blood pressure and reducing the risk of cardiovascular events, cardiovascular mortality and all-cause mortality [79].

In the general population, a systematic review and meta-analysis of 104 studies involving 2,755,719 patients without baseline CKD found that higher dietary potassium intake was linked to a lower risk of developing CKD [80]. Similarly, a large prospective, registry-based observational cohort study of 317,162 patients without CKD reported that higher potassium intake was associated with lower risk of developing CKD [81].

However, in patients with established CKD, similar evidence supporting the use of salt substitutes or liberalized dietary potassium intake is lacking. Nonetheless, there is growing skepticism about the validity of the traditional approach of routine dietary potassium intake in this population.

A cross-sectional study including 95 patients with CKD and 117 patients with ESKD undergoing maintenance HD n found no association between higher dietary potassium intake and hyperkalemia in either group [82]. This aligns with findings from a retrospective cohort study of 8043 ESKD patients on maintenance HD, which similarly reported no increased risk hyperkalemia or all-cause mortality with higher dietary potassium intake [83].

**Table 3:** Studies associating Higher Potassium Intake with CKD outcomes and Mortality *.

Study	Population	Outcomes
Mirmiran et al. 2018 [84]	1780 participants in the Tehran Lipid and Glucose study and followed up for 6.3 yr	No difference in risk of incident CKD
Kieneker et al. 2016 [85]	5315 Dutch participants aged 28 to 75 yr in the PREVEND study and followed up for a median of 10.3 yr	Reduced risk of incident CKD
Kelly et al. 2021 [80]	Meta-analysis of 104 studies involving 2,755,619 patients without baseline CKD	Reduced risk of incident CKD
He et al. 2016 [86]	3939 participants aged 21–74 yr with CKD (GFR 20–70 mL/min per 1.73 m^2^) in the CRIC study	Increased risk of CKD progression No difference in risk of death
>Araki et al. 2015 [87]	>623 Japanese patients with diabetes and eGFR ≥ 60 mL/min per 1.73/m^2^ enrolled between 1996–2003 and followed up until 2013	>Reduced risk of CKD progression Slower rate of annual eGFR decline
>Smyth et al. 2014 [88]	>Post hoc analysis of ONTARGET and TRANSCEND studies; >30,000 patients from 18 countries with vascular disease or diabetes with end-organ damage	>Reduced risk of CKD progression
Kim et al. 2019 [89]	1821 participants aged 20–75 yr with CKD G1–G5 (non-dialysis) in the KNOW-CKD study	Reduced risk of CKD progression
Smyth et al. 2016 [90]	544,635 participants in the NIH-AARP Diet and Health Study, aged 51–70 yr	Reduced risk of ESKD and renal mortality
Noori et al. 2010 [91]	224 chronic HD patients from the NIED Study	Increased risk of death when comparing extremes of dietary potassium intake
Bernier-Jean et al. 2021 [83]	8043 participants in the DIET-HD study with ESKD on maintenance HD	No difference in risk of death
Neal et al. 2021 [78]	Open-label, cluster-randomized trial involving 600 villages and 20,995 individuals in rural China with a history of stroke or aged ≥ 60 years old with poorly controlled blood pressure	Reduced risk of stroke, major adverse cardiovascular events and all-cause mortality
Yin et al. 2022 [79]	Meta-analysis of 21 studies involving 31,949 patients evaluating the effect of salt substitutes on clinical outcomes	Reduced blood pressure Reduced cardiovascular events, cardiovascular mortality and all-cause mortality
Leonberg-Yoo et al. 2017 [92]	Post hoc analysis of MDRD study 812 patients aged 15–70 yr with CKD G2–G4	Reduced risk of death
Eisenga et al. 2016 [93]	Prospective cohort of 705 stable kidney transplant recipients	Reduced risk of graft failure and death

CRIC, Chronic Renal Insufficiency Cohort; KNOW-CKD, Korean Cohort Study for Outcome in Patients with CKD; MDRD, Modification of Diet in Renal Disease; NIED, Nutritional and Inflammatory Evaluation in Dialysis; NIH-AARP, National Institutes of Health–American Association of Retired Persons; ONTARGET, Ongoing Telmisartan Alone and in Combination with Ramipril Global Endpoint Trial; PREVEND, Prevention of renal and vascular end-stage disease; TRANSCEND, Telmisartan Randomized Assessment Study in ACE Inhibitor Intolerant Subjects with Cardiovascular Disease. * Adapted from Clase et al. 2020 KDIGO Controversies Conference on Potassium Homeostasis and Management of Dyskalemias in Kidney Diseases [8].

### 3.2. Dietary Patterns vs. Nutrient Focus

Numerous publications have highlighted the potential cardio-renal-metabolic benefits of various plant-based dietary approaches, as well as the individual components of these diets, particularly dietary sodium and potassium intake [80,94]. This growing body of evidence supporting the potential benefits of plant-based diets was acknowledged as a practice point in the 2024 iteration of the KDIGO Clinical Practice Guidelines for CKD, encouraging the medical community to advocate for healthier dietary practices with a higher consumption of plant-based foods compared to animal-based foods and a lower consumption of ultra-processed foods amongst patients with CKD [13]. However, the optimal dietary approach for patients with CKD remains unclear. Plant-based diets encompass a range of patterns, including the Dietary Approaches to Stop Hypertension (DASH) diet, the Mediterranean diet, the Plant-Dominant Low-Protein Diet, vegan, vegetarian and whole plant-based diets [95]. Immediate benefits of these diets, with respect to the risk of development of hyperkalemia, include (i) reduced net acid production compared to typical Western diets (which contributes to intracellular movement of potassium) and (ii) improvement of constipation (a known contributor to hyperkalemia, particularly in patients with advanced CKD). Notably, small RCTs have demonstrated that plant-based diets can be as effective as oral sodium bicarbonate supplementation in managing CKD-related metabolic acidosis, without increasing the risk of hyperkalemia, even in patients with more advanced CKD [31,32,33,34]. Additionally, plant-based diets may also have favorable effects on phosphorus metabolism in patients with CKD [96]. A meta-analysis of seven observational studies involving 15,285 participants with CKD further supports the association between plant-based diets and reduced mortality in this population. [94].

#### 3.2.1. Dietary Approaches to Stop Hypertension

The original DASH study by Appel et al. published in NEJM in 1997, was a randomized controlled trial involving 459 participants with systolic blood pressure < 160 mmHg and diastolic blood pressure of 80–95 mmHg. This study compared combination diet, rich in fruits, vegetables, whole grains and low-fat dairy products with reduced saturated and total fat (referred to as the DASH diet) against a control diet [97]. The DASH diet was shown to significantly lower blood pressure, and its findings were subsequently corroborated by numerous studies that followed, leading to its widespread adoption for the prevention and management of hypertension. Additional evidence has demonstrated its cardiorenal benefits, including reduced risk of kidney disease, cardiovascular events, and all-cause mortality (Table 4) [98,99,100,101,102,103]. Further research has shown that the DASH diet has a lower dietary acid load compared to typical Western diets [104,105]. Although the original DASH study did not evaluate the impact of sodium intake, the follow-up DASH-Sodium trial demonstrated that combining the DASH diet with reduced dietary sodium intake produced additive effects in lowering blood pressure [106].

#### 3.2.2. Mediterranean Diet

The Mediterranean Diet is characterized by a high intake of plant-based foods, including cereals, fruits, herbs, legumes, nuts and vegetables, limited consumption of red meat and moderate intake of fish/seafood, eggs, white meat and dairy products. Its principal source of fat is olive oil. This dietary pattern offers numerous benefits for patients with CKD, including improved control of cardiovascular risk factors and a reduced risk of incident CKD and cardiovascular disease (Table 5) [112,113,114]. The 2020 Kidney Disease Outcomes Quality Initiative (KDOQI) Clinical Practice Guidelines for Nutrition in CKD recommend the Mediterranean diet for adults with CKD G1–G5 not on dialysis, as well as for kidney transplant, to improve lipid control [115]. Additionally, for patients with CKD G1–G4, increased consumption of fruit and vegetable is advised to help reduce body weight, blood pressure, and dietary acid load [115]. These recommendations were echoed in the 2024 KDIGO guidelines for CKD, which advocate for a plant-based Mediterranean diet in conjunction with a lipid-modifying therapy to reduce cardiovascular risk [13]. Several review articles, including those by Perez-Torres et al. and D’Alessandro et al., provide practical strategies for adapting the Mediterranean diet to different stages of CKD [116,117].

### 3.3. Food Sources, Bioavailability of Potassium and Food Preparation Methods

When guiding patients towards accessible sources of dietary potassium, many plant-based foods such as bananas, citrus juices, melons, avocados, potatoes and tomatoes, stand out as rich potassium sources. These foods can be easily incorporated into a healthy diet for patients with CKD, offering nutritional benefits beyond potassium content. Notably, severe of these foods are high in carbohydrates, which may stimulate insulin release and help mitigate postprandial increases in plasma potassium concentrations. In contrast, animal-based food typically lacks carbohydrates and may lead to greater elevations in plasma potassium following consumption [1].

Traditional dietary guidelines for CKD have often restricted the consumption of vegetables and whole grain products due to their relatively high potassium and phosphorus content. Emerging evidence demonstrates that the bio-availability of dietary potassium (and phosphate) in plant-based foods is generally <50%, compared to nearly 100% from ultra-processed foods (rich in potassium additives), meats, dairy products, juices and salt substitutes made with potassium chloride [122,123]. Processed foods, particularly those containing additives, contribute significantly to dietary potassium and phosphate loads in patients with CKD [123,124,125,126]. In addition, a recent animal model study comparing a high KHCO_3_ diet to a high KCl diet demonstrated that dietary anions, in particular HCO_3_^−^, have a potential role in the prevention of hyperkalemia by increasing urinary potassium excretion through various mechanisms [127,128]. As such, dietary counselling for patients with CKD should go beyond macronutrients quantity and food preparation methods and instead emphasize the importance of nutrient sources. This sentiment is reflected in the latest 2024 KDIGO guidelines for CKD that encourages the adoption of healthier diets with a higher consumption of plant-based foods and lower consumption of ultra-processed foods [13].

A systematic review of 65 articles demonstrated that while all food preparation techniques can reduce potassium content, methods involving water, such as boiling or soaking tend to result in greater reductions in potassium (Table 6) [129]. However, this review had notable limitations, including significant heterogeneity in study designs with few studies identified for certain cooking techniques. Martinez-Pineda et al. also demonstrated that frozen products achieved greater reductions in potassium content compared to fresh products [130].

### 3.4. Clinical Trials and Guidelines

#### 3.4.1. Upcoming Clinical Trials

An ongoing randomized trial with a two-period cross-over design involving 30 patients with CKD and an estimated glomerular filtration rate of 15–45 mL/min/1.73 m^2^ aims to generate high-quality evidence to support or refute current recommendations for dietary potassium restriction in patients with CKD Stage G3b to G4 [131]. At time of publication, the study is still actively recruiting patients.

In parallel, a recent proof-of-concept study by Liebman et al. explored the potential benefits of a 15-day whole-food, plant-based nutrition education program in patients with CKD Stage G3 or G4 [132]. Although the observed reductions in blood pressure were not statistically significant, the intervention group demonstrated improvements in body mass, total cholesterol, low-density lipoprotein and high-density lipoprotein without increased risk of hyperkalemia compared to controls [132]. These findings are promising but require validation in larger, more diverse populations with a longer follow-up duration to confirm the efficacy and safety of such novel dietary interventions in CKD management.

#### 3.4.2. Current Clinical Practice Guidelines

A review by Xu et al. highlighted inconsistencies across major hypertension and CKD guideline regarding dietary potassium restriction and the use of potassium-enriched salt substitutes. While some guideline supports their use for cardiovascular risk reduction, there remains a widely accepted caution against liberation of potassium restriction and use of salt substitutes in individuals with advanced CKD due to the potential risk of hyperkalemia (Table 7 and Table 8) [133].

## 4. Our Recommendations

To further optimize cardiovascular outcomes in patients with CKD, we recommend an individualized approach to liberalizing dietary potassium intake, with a focus on substituting ultra-processed and animal-based diets with minimally processed, plant-based food sources. This should be undertaken in consultation with a renal dietitian or accredited nutritional professional, accompanied by close biochemical monitoring, particularly in patients with more advanced CKD, eGFR < 30 mL/min/1.73 m^2^, with either (i) a history of hyperkalemia and/or (ii) use of RAASi (including ACEi/ARB/nsMRA/MRA). Other notable risk factors that contribute to risk of developing hyperkalemia include presence of diabetes, concomitant heart failure, and over-the-counter non-steroid anti-inflammatory drug use. In stable patients without existing hyperkalemia, we would suggest routine monitoring of serum potassium in these patients at least 3–4 times per year.

For individuals identified with hyperkalemia, consideration should be made to raise thresholds for urgent intervention (i.e., warranting accident and emergency visit and/or inpatient hospitalization) for mild or even moderate hyperkalemia (serum K^+^ < 6.0 mmol/L) in well, asymptomatic individuals without acute kidney injury. Our rationale is that majority of these patients can be assessed and managed safely as outpatients. Our goal should be to implement an affordable strategy for management of hyperkalemia that allows the maintenance of other therapies that either delay CKD progression or mitigate cardiovascular risk.

In patients with chronic hyperkalemia, early use of novel potassium binders can not only facilitate the continuation and optimization of RAASi but can also prevent development of moderate to severe hyperkalemia (serum potassium ≥ 5.6 mmol/L) and its attendant healthcare costs. In these patients requiring titration of medication doses, we would suggest regular monitoring of serum potassium at 2–4 weekly intervals depending on CKD stage, current eGFR and concomitant medications until stable dose of medications achieved to maintain normokalaemia. Subsequent monitoring can then be done at least 3–4 times per year.

Here, we have suggested a multi-pronged approach to management of varying degrees of severity of hyperkalemia (Table 9).

The potential benefits for recommending the routine use of potassium enriched salt substitutes in patients with CKD are less certain given the lack of evidence supporting such practices in our CKD population. Therefore, at present this should only be cautiously considered in individuals with compelling cardiovascular risk or uncontrolled hypertension, if serum potassium remains in the normal range following implementation of the above measures. After introduction of salt substitutes, an individualized monitoring strategy should be employed, considering the patient’s comorbidities and concomitant medications.

## 5. Future Directions

In the potassium space, more randomized, controlled studies are required to examine the effects of liberal intake on clinically relevant outcomes, as well as to assess the safety of conservative versus aggressive management approaches to serum levels and potential implications for healthcare costs. The scale, complexity, and financial demands of such studies warrant thoughtful collaboration and investment from a broad spectrum of stakeholders, including clinicians, researchers, industry partners and policy makers to ensure meaningful progress for this particularly vulnerable population.

## 6. Conclusions

The paradigm shift in our approach to management of potassium in patients with CKD can potentially translate to improved patient outcomes in terms of CKD progression and major adverse cardiovascular events. However, the onus is on the medical community to advocate for (i) liberalization of dietary potassium restrictions, particularly in patients with early CKD, (ii) increasing consumption of plant-based foods (and consequently reduction of animal-based and ultra-processed food sources), (iii) optimization of RAASi prescription (with/without prescription of novel potassium binding resins) and (iv) furthering research to identify more generalizable, preferably population-wide, dietary interventions that are efficacious and safe for the CKD population to mitigate their significant residual risks of adverse outcomes, particularly cardiovascular disease-related.

## Figures and Tables

**Table 1:** Studies associating Discontinuation/Dose Reduction of RAASi with Adverse Cardio-Renal Outcomes.

Dose Reduction of RAASi		
Study	Population	Outcomes
Svensson et al. 2024 [25]	Retrospective multination cohort study involving 40,059 patients (Germany, Spain, Sweden and UK) with CKD and/or heart failure with a history of RAASi related hyperkalemia	Higher risk of 6-month hospitalization
Svensson et al. 2024 [26]	Retrospective cohort study involving 28,613 patients (Sweden and Japan) with CKD and/or heart failure with a history of RAASi related hyperkalemia	Longer hospital length of stay/hospitalized days
Kanda et al. 2023 [23]	Retrospective cohort study involving 21,508 patients (Japan and USA) with CKD and/or heart failure with a history of RAASi related hyperkalemia who either (i) discontinued or (ii) down-titrated their RAASi compared to patients who maintained/up-titrated their RAASi dose following the hyperkalemia episode	Both discontinuation and dose reduction of RAASi was associated with higher risk of cardiorenal composite outcome (heart failure emergency room visits/hospitalizations, progression to ESKD)
**Discontinuation of RAASi**		
**Study**	**Population**	**Outcomes**
Fu et al. 2021 [21]	Retrospective cohort study involving 10,254 patients (Sweden) who developed advanced CKD, eGFR < 30 mL/min/1.73 m^2^ stratified by continuation of RAASi	Higher risk of MACE and all-cause mortality Lower risk of ESKD
Leon et al. 2022 [19]	Retrospective cohort study involving 78,490 patients (Canada) with CKD with a history of RAASi related hyperkalemia	Higher risk of all-cause mortality, CV mortality and increased risk of dialysis initiation
Yang et al. 2023 [24]	Prospective, population-based cohort study involving 10,400 patients (Hong Kong) with type 2 diabetes and advanced CKD, eGFR < 30 mL/min/1.73 m^2^ stratified by continuation of RAASi	Higher risk of MACE, heart failure and ESKD
Bhandari et al. 2022 [22]	Multi-center, randomized, controlled trial involving 411 patients with advanced CKD, eGFR < 30 mL/min/1.73 m^2^ comparing discontinuation vs. continuation of RAASi	No difference in primary outcome of eGFR at 3 years No difference in key secondary outcomes of CKD progression, ESKD Reported similar incidence of adverse events with respect to cardiovascular events and deaths, however not adequately powered to detect significant differences in either outcome MACE, all-cause mortality

**Table 2:** Studies associating Lower Potassium Intake with CKD outcomes and Mortality *.

Study	Population	Outcomes
Suenaga et al. 2025 [47]	Prospective cohort study involving 4314 participants (Japan) with CKD	Higher risk of CKD progression
Ma et al. 2022 [48]	Meta-analysis of 6 studies involving 10,709 healthy adult participants * (namely the Health Professionals Follow-up Study, the Nurses’ Health Study, the Nurses’ Health Study II, the Prevention of Renal and Vascular End-Stage Disease Study and the Trials of Hypertension Follow-up Studies)	Higher risk of cardiovascular disease; dose-dependent
Narasaki et al. 2022 [50]	Retrospective cohort study involving 37,893 participants stratified according to normal and impaired kidney function	Higher risk of all-cause mortality irrespective of kidney function Higher risk of all-cause mortality in patients with CKD when paired with high protein, low fiber and high phosphorus intake
Narasaki et al. 2021 [49]	Prospective cohort study involving 415 participants (USA) with ESKD on maintenance HD	Higher risk of all-cause mortality

* Due to the methodology of this study (e.g., exclusion of patients with CKD from PREVEND study), its study population generally consisted of healthy White participants and therefore generalizability of results to other racial/ethnic groups or to patients with specific conditions like CKD is not possible.

**Table 4:** Benefits of DASH Diet.

Study	Population	Outcomes
Saneei et al. 2014 [99]	Systematic review and meta-analysis of 17 randomized, controlled studies involving 2561 participants	DASH diet significantly reduced systolic blood pressure by 6.74 mmHg and diastolic blood pressure by 3.54 mmHg, particularly amongst participants with hypertension
Siervo et al. 2015 [100]	Systematic review and meta-analysis of 20 randomized, controlled studies involving 1917 participants	DASH diet significantly reduced systolic blood pressure, diastolic blood pressure and total cholesterol, particularly amongst participants with increased cardiometabolic risk; these improvements in cardiovascular risk factors predicted ~13% reduction in the 10-year Framingham risk score for cardiovascular disease
Rebholz et al. 2016 [107]	Prospective cohort study involving 14,882 participants from the Atherosclerosis Risk in Communities (ARIC) Study with baseline eGFR ≥ 60 mL/min/1.73 m^2^	DASH diet associated with lower risk of incident CKD High red meat and processed meat intake (animal-based protein) was associated with higher risk of CKD whereas high nuts, legumes and low-fat dairy product intake (vegetable and dairy sources of protein) was associated with lower risk for CKD
Lee et al. 2017 [108]	Retrospective cohort study involving 2408 elderly participants (Korea) NOTE mean age 72.4 ± 5.1 years old; only 23.8% had diabetes mellitus and 13.9% had CKD	DASH diet associated with lower risk of CKD
Maroto-Rodriguez et al. 2025 [109]	Prospective cohort study involving 106,870 participants from the UK Biobank	DASH diet associated with lower risk of CKD
Hu et al. 2021 [110]	Prospective cohort study involving 2403 participants with eGFR 20–70 mL/min/1.73 m^2^ from the Chronic Renal Insufficiency Cohort (CRIC) Study cohort	DASH diet associated with lower risk of CKD progression and all-cause mortality
Banerjee et al. 2019 [111]	Retrospective cohort study involving 1110 participants (USA) with HTN and CKD, eGFR 30–59 mL/min/1.73 m^2^ NOTE study population included participants of Third National Health and Nutritional Examination Survey conducted from 1988 to 1994 before the DASH study was published	DASH diet associated with lower risk of ESKD, particularly in patients with diabetes Mediation analyses identified potassium and magnesium intake as strong mediators and dietary acid load and protein intake as partial mediators of the observed association between the DASH diet and ESKD
Soltani et al. 2020 [103]	Systematic review and meta-analysis of 13 prospective cohort studies involving 1,240,308 participants	DASH diet associated with lower risk of all-cause mortality

**Table 5:** Benefits of Mediterranean Diet.

Study	Population	Outcomes
Hansrivijit et al. 2020 [118]	Systematic review and meta-analysis of 4 studies involving 8467 participants	Mediterranean diet reduced risk of incident CKD
Podadera-Herreros et al. 2022 [119]	Randomized, controlled trial involving 1002 participants with type 2 diabetes mellitus, coronary heart disease and eGFR ≥ 30 mL/min/1.73 m^2^ from the CORDIOPREV study cohort; comparing Mediterranean diet vs. a low-fat diet	Mediterranean diet reduced rate of eGFR decline, particularly in patients with mildly impaired eGFR 60–89 mL/min/1.73 m^2^
Maroto-Rodriguez et al. 2025 [109]	Prospective cohort study involving 106,870 participants from the UK Biobank	Alternate Mediterranean diet associated with lower risk of CKD
Hu et al. 2021 [110]	Prospective cohort study involving 2403 participants with eGFR 20–70 mL/min/1.73 m^2^ from the Chronic Renal Insufficiency Cohort (CRIC) Study cohort	Alternate Mediterranean diet associated with lower risk of CKD progression and all-cause mortality
Papadaki et al. 2020 [120]	Systematic review and meta-analysis of 57 studies involving 36,983 participants	Mediterranean diet improved metabolic syndrome components/cardiovascular risk factor control in addition to reduced risk of cardiovascular disease and stroke
Kwon et al. 2024 [121]	A pilot, randomized, controlled trial with crossover design involving 50 participants (Korea) with CKD G3–G4; comparing Mediterranean Proper Optimal Balance (MEDi-POB) diet vs. control for 12 weeks	MEDi-POB diet significantly lowered dietary sodium intake, increased total CO_2_ levels (indicating reduced dietary acid load) and non-significant increase in dietary potassium intake; there were no significant changes in kidney function, serum and urinary potassium levels—pilot study indicating safety of Mediterranean diet in patients with CKD G3–G4

**Table 6:** Food Preparation Techniques and Efficacy in Potassium Reduction.

Cooking Technique	Level of Potassium Reduction	Suggested Food Group (s)
Cooking in water	Highest reduction in potassium (32–71%)	Most food groups except fish/seafood, cereal and derivates
Soaking (and other techniques involving water)	Good efficacy in reduction in potassium (17–60%)	Cruciferous vegetables Leafy vegetables Legumes
Steam Cooking	Modest efficacy in reduction in potassium (11–38%)	Most food groups except cereal and derivates *
Dry Heat Cooking	Lowest efficacy in reduction in potassium	Reserve for use in fruit and derivates, tubers and roots

* NOTE interpret with caution because data/results drawn from a single study.

**Table 7:** Dietary Potassium Restriction in CKD Management Guidelines.

Organization	Year	Recommendation
International Guidelines		
Kidney Disease: Improving Global Outcomes	2024	3.3.2 Use renal dieticians or accredited nutrition providers to educate people with CKD about dietary adaptations regarding sodium, phosphorus, potassium, and protein intake, tailored to their individual needs, and severity of CKD and other comorbid conditions 3.11.5 In early stages of CKD, high intake of foods naturally rich in potassium appears to be protective against disease progression, and dietary restriction of foods naturally containing potassium, such as fruits and vegetables, may be harmful to cardiac health; therefore, such restriction is not endorsed. 3.11.5.1 Implement an individualized approach in people with CKD G3–G5 and emergent hyperkalemia that includes dietary and pharmacologic intervention and takes into consideration associated comorbidities and quality of life (QoL). Assessment and education through a renal dietician or an accredited nutrition provider are advised
Regional Guidelines		
Europe Renal Association	2014	Nil recommendations
Caring for Australian & New Zealanders with Kidney Impairment	2013	Early CKD patients with persistent hyperkalemia restrict their dietary potassium intake with the assistance of an appropriately qualified dietician.
National Guidelines		
Kidney Disease Outcomes Quality Initiative	2020	In adults with CKD 3–5D or post transplantation, it is reasonable to adjust dietary potassium intake to maintain serum potassium within normal range (OPINION) In adults with CKD 3–5D (2D) or post transplantation (OPINION) with either hyperkalemia or hypokalemia, we suggest that dietary or supplemental potassium intake be based on patient’s individual needs and clinician judgement.

**Table 8:** Potassium-Enriched Salt Recommendations in CKD Management Guidelines.

Organization	Year	Recommendation
International Guidelines		
Kidney Disease: Improving Global Outcomes	2024	Nil recommendation
Kidney Disease: Improving Global Outcomes	2021	The Dietary Approaches to Stop Hypertension (DASH)–type diet or use of salt substitutes that are rich in potassium may not be appropriate for patients with advanced CKD or those with hyporeninemic hypoaldosteronism or other causes of impaired potassium excretion because of the potential for hyperkalemia.
Regional Guidelines		
Europe Renal Association	2014	Nil recommendations
Caring for Australian & New Zealanders with Kidney Impairment	2013	Patients with CKD should not use salt substitutes that contain high amounts of potassium salt. Early CKD patients with persistent hyperkalemia restrict their dietary potassium intake with the assistance of an appropriately qualified dietician.
National Guidelines		
Kidney Disease Outcomes Quality Initiative	2020	Nil recommendations

**Table 9:** A Modern Approach to Management of Hyperkalemia in CKD.

Degree of Hyperkalemia	Recommendations
Mild Serum K^+^ 5.1–5.4 mmol/L	Address correctable factors - Review of concomitant medications (Avoid non-steroid anti-inflammatory drugs; review indications and consider alternatives for other concomitant medications associated with increased risk of hyperkalemia include β-adrenergic receptor blockers, digitalis glycoside, heparin, calcineurin inhibitors, trimethoprim and pentamidine) - Referral to dietician for assessment of dietary potassium intake and education focused on the least obtrusive dietary modifications and food preparation methods - Consider use of diuretics, particularly in patients with peripheral edema and/or suboptimal blood pressure control - Consider sodium bicarbonate supplementation to maintain serum bicarbonate > 22 mmol/L Consider early initiation of potassium binding resins, preferably PO SZC or Patiromer (although PO SPS remains a viable alternative if other options are not available), especially if patient is planned for initiation or uptitration of dose of RAASi to delay CKD progression Avoid reduction/discontinuation of RAASi
Moderate Serum K^+^ 5.5–5.9 mmol/L	Address correctable factors as detailed above Assessment of symptoms and/or complications related to hyperkalemia - Assessment and monitoring might include repeat biochemical testing and should include an electrocardiogram Initiation of potassium binding resins, preferably PO SZC or Patiromer (although PO SPS remains a viable alternative if other options are not available) - Example of an initial dose PO SZC 10 g TDS for up to 48 h, followed by maintenance dose from 5 g EOD to maximum dose up to 15 g once daily; NOTE we should aim for the lowest effective maintenance dose that can maintain normokalaemia - Initial repeat testing of serum potassium should be arranged within 3–7 days; earlier date is preferred should RAASi be continued - Subsequent regular monitoring of serum potassium levels every 2–4 weeks while ongoing titration of medication dose Consider reduction/discontinuation of RAASi only if nil correctable factors identified and persistent moderate hyperkalemia despite maximum dose of potassium binding resins prescribed - Patients should be counselled regarding potential implications of RAASi dose reduction/discontinuation with regards to CKD and cardiovascular outcomes and this decision should be reviewed at subsequent follow-up visits if patient’s condition allows
Severe/Life-threatening Serum K^+^ ≥ 6.0 mmol/L	Urgent assessment and intervention required in accident and emergency/hospital - Assessment and monitoring should include repeat biochemical testing and urgent electrocardiogram/telemetry monitoring - Treatment for hyperkalemia including (i) administration of intravenous insulin with 50% dextrose, (ii) administration of IV calcium gluconate and (iii) prescription of potassium binding reins, preferably PO SZC or Patiromer - Temporary suspension of RAASi, if any Initiation of maintenance potassium binding resins, after resolution of acute hyperkalemia episode, preferably PO SZC or Patiromer (although PO SPS remains a viable alternative if other options are not available) - Initial repeat testing of serum potassium should be arranged within 3–7 days; earlier date is preferred should RAASi be resumed after normalization of serum potassium - When RAASi are temporarily suspended, patients should be reminded to review decision during repeat testing of serum potassium to see when it is safe to resume RAASi - Subsequent regular monitoring of serum potassium levels every 2–4 weeks while ongoing titration of medication dose Consider reduction/discontinuation of RAASi only if nil correctable factors identified and persistent moderate hyperkalemia despite maximum dose of potassium binding resins prescribed - Patients should be counselled regarding potential implications of RAASi dose reduction/discontinuation with regards to CKD and cardiovascular outcomes and this decision should be reviewed at subsequent follow-up visits if patient’s condition allows

## Data Availability

Not applicable.

## Supplementary Materials

The following supporting information can be downloaded at: https://www.mdpi.com/article/10.3390/jcm14248917/s1, Table S1: Summary of studies on the efficacy of serum potassium reduction with Sodium Zirconium Cyclosilicate and Patiromer.

## Abbreviations

The following abbreviations are used in this manuscript:

AKI	Acute Kidney Injury
ACEi	Angiotensin-converting enzyme inhibitors (ACEi)
ARB	Angiotensin-receptor blockers
ASDN	Aldosterone-sensitive distal nephron
CKD	Chronic Kidney Disease
CONFIDENCE	Finerenone with Empagliflozin in Chronic Kidney Disease and Type 2 Diabetes
DASH	Dietary Approaches to Stop Hypertension
DM	Diabetes Mellitus
eGFR	Estimated glomerular filtration rate
ENaC	Epithelial Na^+^ channels
ESKD	End Stage Kidney Disease
FIDELIO-DKD	Effect of Finerenone on Chronic Kidney Disease Outcomes in Type 2 Diabetes
GFR	Glomerular filtration rate
HD	Hemodialysis
KDIGO	Kidney Disease: Improving Global Outcomes
MACE	Major adverse cardiovascular events
MRA	Mineralocorticoid receptor antagonists
NCC	Na^+^/Cl^−^ cotransporter
NHE	Sodium-hydrogen exchanger
NKCC	Na^+^/K^+^/2Cl^−^ cotransporter
nsMRA	Non-steroid mineralocorticoid antagonists
PREVEND	Prevention of Renal and Vascular End-Stage Disease Study and the Trials of Hypertension Follow-up Studies
RAASi	Renin-angiotensin-aldosterone system inhibitor
ROMK	Renal outer medullary potassium channels
SGLT2i	Sodium glucose transporter 2 inhibitors
uACR	Urine albumin creatinine ratio
