# Supplementary material for: Rethinking Potassium Management in Chronic Kidney Disease—A Modern Approach
Source: J Clin Med. 2025 Dec 17;14(24):8917. doi: 10.3390/jcm14248917 (PMC12733500; doi:10.3390/jcm14248917)
Supplement: Supplementary file 1 [file jcm-14-08917-s001.zip › jcm-4028387-supplementary.pdf]

Table S1: Summary of studies on the efficacy of serum potassium reduction with Sodium Zirconium Cyclosilicate and Patiromer

	Study design	Population	Baseline RAAS-i	Intervention	Outcomes	Results
**Sodium Zirconium Cyclosilicate (SZC)**						
ZS-003 (Packham DK, 2015 Jan 15)	Phase 3, multi-center (65 sites), RCT, (2-phases), placebo-controlled.	754 ambulatory patients with serum K^+ 5.1-6.5 mmol/L (initial phase).<br><br>543 patients who achieved normal serum K^+ (3.5-5.0 mmol/L), entered randomization for maintenance phase.	Placebo (63.9%)<br>SZC 1.25g (70.8%)<br>SZC 2.5g (68.8%)<br>SZC 5g (63.1%)<br>SZC 10g (67.1%)	Initial phase<br>SZC 1.25g, 2.5g, 5g, 10g, or placebo; administered tid with meal.<br><br>Maintenance phase<br>SZC<br>Subject in the placebo arm during acute phase was randomized to SZC 1.25g, 2.5g daily.<br>Subjects who received active drug was randomized to placebo or maintaining at same dose of SZC.	Initial phase:<br>Difference in exponential rate of change in the mean serum K^+ during the first 48 hours<br><br>Maintenance phase<br>Difference in mean serum K^+ during the 12-day treatment interval	Initial phase:<br>Serum K^+ reduction in 1 hour<br>SZC 10g: -0.11 mmol/L *(95% CI -0.17 to -0.05)*<br>Placebo: +0.01 mmol/L *(95% CI -0.05 to 0.07)*<br><br>Serum K^+ reduction at 48H<br>ZS 2.5g: -0.46mmol/L *(95% CI -0.53 to -0.39)*<br>ZS 5g: -0.54 mmol/L *(95% CI -0.62 to -0.47)*<br>ZS 10g: -0.73 mmol/L *(95% CI -0.82 to -0.65)*<br>Placebo: -0.25 mmol/L *(95% CI -0.32 to -0.19)*<br>*(p<0.001 for all comparison)*<br><br>Maintenance phase at day 21<br>SZC 10g, 5g: greater exponential decrease in serum K^+ (p=0.0083 and p<0.0001).<br><br>SCZ 10g: longer normokalaemic duration (10.2 days) vs placebo (8.2 days)<br>SZC 5g: longer normokalaemic duration (9.0 days) vs placebo (6.0 days)
ZS-004 (HARMONIZE) (Mikhail Kosiborod, et al., 2014)	Phase 3, multi-center (44 sites), RCT, placebo-controlled.	258 ambulatory patients with serum K^+ > 5.1 mmol/L (initial phase).<br><br>237 patients who achieved normal serum K^+ (3.5-5.0 mmol/L), entered randomization for maintenance phase.	Placebo (71.8%)<br>SZC 5g (73.3%)<br>SZC 10g (70.6%)<br>SZC 15g (58.9%)	Initial phase (open-label)<br>SZC 10g tid for 48 hours<br><br>Maintenance phase (double-blind)<br>SZC 5, 10, 15g daily vs placebo for 28 days	Comparison of mean serum K^+ between placebo and each treatment group during day 8-29.	Initial phase (SZC 10g tid):<br>-0.2 mmol/L at 1 hour (95% CI, -0.3 to -0.2)<br>-0.4 mmol/L at 2 hours (95% CI, -0.5 to -0.4)<br>-0.5 mmol/L at 4 hours (95% CI, -0.6 to -0.5)<br>-0.7 mmol/L at 24 hours (95% CI, -0.7 to -0.6; -12%)<br>-1.1 mmol/L at 48 hours (95% CI, -1.1 to -1.0; -19%)<br><br>Serum K^+ declined from 5.6 mmol/L (baseline) to 4.8 mmol/L (at 48 hours).<br><br>Median time to normalization of serum K^+: 2.2 hours (IQR 1.0 to 22.3).<br><br>Mean serum K^+, maintenance phase:<br>SZC 5g: 4.4 mmol/L (95% CI, 4.3 to 4.5)<br>SZC 10g: 4.5 mmol/L (95% CI, 4.4 to 4.6)<br>SZC 15g: 4.8 mmol/L (95% CI, 4.6 to 4.9)<br>Placebo: 5.1 mmol/L (95% CI, 5.0 to 5.2)
ZS-005 (Bruce S Spinowitz, 2019 June 7)	Phase-3, multi-center, RCT, open-	751 patients with serum K^+ ≥ 5.1	RAASi (65%)<br>ACEi (45%)<br>ARB (21%)	Correction phase:<br>SZC 10g tid for 72 hours	Correction phase<br>Serum K^+ 3.5 – 5.0 mmol/L at 72 hours	Proportion of patients achieved normal serum K^+ during correction phase<br>At 24 hours: 66.0%

	label, single-arm	mmol/L (Correction phase).<br><br>746 patients who achieved normal serum K^+ (3.5-5.0 mmol/L) entered maintenance phase.<br><br>74% eGFR < 60 ml/min/1.73 m^2	MRA (6%)	Maintenance phase: SZC 5g, daily, titrated in 5-g increment or decrements (maximum 15g daily, minimum 5g every other day) to maintain serum K^+ 3.5-5.0 mmol/L.	Maintenance phase Serum K^+ < 5.1 and 5.5 mmol/L over months 3-12	At 48 hours: 75.3%<br>At 72 hours: 77.9%<br><br>99.9% of patients achieved serum K^+ 3.5 – 5.5 mmol/L at 72 hours.<br><br>Proportion of patients achieved serum K^+ 3.5 – 5.1 mmol/L during maintenance phase (over 12 months): 88%.<br><br>Mean daily SZC dose received was 7.2g (SD 2.6) administered over a mean 286 days.
DIALYZE (Fishbane S F. M.-S., 2019 Sep)	Phase-3b, multi-center (54 sites), double-blind, placebo-controlled.	196 adult haemodialysis patients with pre-dialysis hyperkalaemia (Serum K^+ > 5.4 mmol/L after the long interdialytic interval, and serum K^+ > 5.0 mmol/L after the short interval)		SZC 5g (5-15g) qd on non-dialysis day to maintain the long interdialytic interval serum K^+ 4.0-5.0 mmol/L.	Proportion of "responder" (maintained serum K^+ 4-5 mmol/L) during >3 HD treatment after the long interdialytic interval and those who did not require rescue therapy (serum K^+ > 6.0 mmol/L)	"Responder" with serum K^+ 4.0-5.0 mmol/L:<br>SZC 41.2% vs Placebo 1.0%; OR 68.8; 95% CI, 10.9to 2819. (*P*<0.01).<br><br>Rescue therapy to reduce serum K^+:<br>SZC 2.1% vs Placebo 5.1%
DIALIZE-Outcomes (Fishbane S, 2025)	Phase-3, multi-center (344 sites), double-blind, placebo-controlled.	2690 adult haemodialysis patients (HD/HDf, 3x/week) with recurrent hyperkalaemia (serum K^+ > 5.5 mmol/L)		SZC 5g (0-15g) to attain pre-dialysis K^+ 4-5 VS placebo on non-dialysis days	Composite of time to first occurrence of any of the 3 (SCD, stroke or arrythmia-related hospitalization); intervention; or ED visit due to arrythmia.	Terminated early due to low event rates and high study medication discontinuation rates.<br><br>Primary outcome: No treatment effect (SZC, 8.8% vs Placebo, 8.9%; HR 0.98; 95% CI, 0.76 to 1.26).<br><br>Maintenance of normokalaemia at 12 months was significantly better with SZC, 74% vs Placebo, 47%, OR 3.36; 95% CI, 2.64 to 4.26).
**Patiromer**						
OPAL-HK (Weir MR & Investigators., 2015 Jan 15)	Multinational, single-blind, two-phase study	243 CKD stage 3 or 4 patients with serum K^+ 5.1 – 6.5 mmol/L and had received a stable dose of one or more RAAS-I for at least 28 days.	RAASi (100%)<br>ACE-I (67-73%)<br>ARB (31-44%)<br>MRA (7-9%)	Initial phase (4-week)<br>Serum K^+ 5.1-5.4 mmol/L: Patiromer 4.2 g BD<br>Serum K^+ 5.5-6.5 mmol/L: Patiromer 8.4g BD.<br><br>Withdrawal phase (8-week) (after achieving normokalaemia): Patiromer same daily dose vs placebo.	Initial treatment phase: Mean change in the serum K^+ level from baseline to week 4.<br><br>Withdrawal phase: Difference between group in the median change of serum K^+ level from the start of week 4,	Initial phase (mean change in serum K^+):<br>−1.01±0.03 mmol/L (95% CI, −1.07 to −0.95; *P*<0.001).<br><br>Change of serum K^+ in mild hyperkalemia:<br>−0.65±0.05 mmol/L (95% CI, −0.74 to −0.55),<br><br>Change of serum K^+ in moderate-to-severe hyperkalaemia:<br>−1.23±0.04 mmol/L (95% CI, −1.31 to −1.16)<br><br>76% of patients achieved target serum K^+ by week 4.<br><br>Withdrawal phase:

						Median serum K^+ increment was 0 in Patiromer group and 0.72 mmol/L in the placebo group, between-group difference of 0.72 mmol/L (95% CI, 0.46 to 0.99; *P*<0.001).<br><br>Higher recurrence of hyperkalaemia (serum K^+ > 5.5 mmol/L) in the placebo group, 60% as compared to patiromer group, 15% (95% CI, 6 to 24) (*P*<0.001).
AMETHYST-DN	Phase 2, multi-center, open-label, dose-ranging, randomized clinical trial	306 outpatients with type 2 diabetes (eGFR, 15 to <60 mL/min/1.73 m^2 and serum K^+ > 5.0 mmol/L)	ACE-i: 43.2-65.4%<br>ARB: 17.9-40.0%<br>MRA: 1.4%	Mild hyperkalaemia (serum K^+ 5.0-5.5)<br>Patiromer 4.2g, 8.4g, 12.6g BD<br>Moderate hyperkalaemia (serum K^+ 5.6-6.0)<br>Patiromer 8.4g BD, 12.6g BD, 16.8g BD.	Mean change in serum K^+ level from baseline to week 4 or prior to initiation of dose titration.	Mean reduction from baseline in serum K^+ for mild hyperkalaemia level at week 4:<br>4.2g BD: 0.35 mmol/L (95% CI, 0.22 to 048)<br>8.4g BD: 0.51 mmol/L (95% CI, 0.38 to 0.64)<br>12.6g BD: 0.55 mmol/L (95% CI, 0.42 to 0.68)<br><br>Mean reduction from baseline in serum potassium for moderate hyperkalaemia at week 4:<br>8.4g BD: 0.87 mmol/L (95% CI, 0.60 to 1.14)<br>12.6g BD: 0.97 mmol/L (95% CI 0.7 to 1.23)<br>16.8g BD: 0.92 mmol/L (95% CI, 0.67 to 1.17)
PEARL-HD (Middleton JP, 2024 Aug 10)	Prospective, randomized, open-label trial.	33 HD (3x/week) patients with at least 2 measured pre-dialysis serum K^+ > 5.5 mmol/L or 1 measured serum K^+ > 6.0 mmol/L over the previous 3 months, with current use of dialysate K^+ 2.0 mmol/L.	RAAS-I: 0 (in Patiromer arm)	Patiromer 8.4g qd (titrate based on serum K^+) vs usual care.<br><br>All patients instructed to limit potassium intake to < 60 mmol/day.	Total number of episodes of serum K^+ > 5.5 mmol/L over 4 weeks.	Number of episodes of serum K^+ > 5.5 mmol/L:<br>13 in Patiromer vs 41 in control group.<br><br>Median number of hyperkalaemia episodes per participants:<br>0 in Patiromer vs 3 in control group, p=0.024)<br><br>Median dose of Patiromer at week 3: 8.4g.

Abbreviations: RCT - randomized controlled trial, HD - haemodialysis, HDF - haemodiafiltration, CKD - chronic kidney disease, eGFR - estimated glomerular filtration rate, RAAS-i - renin angiotensinogen aldosterone inhibitor, ACE-i - angiotensin converting enzyme inhibitor, ARB - angiotensin receptor blockade, MRA - mineralocorticoid receptor antagonist, , tid - three times daily, qd - once daily, BD - twice daily, CI - confidence interval, IQR - interquartile range, OR - odds ratio.
